# Novel brain PET imaging agents: Strategies for imaging neuroinflammation in Alzheimer’s disease and mild cognitive impairment

**DOI:** 10.3389/fimmu.2022.1010946

**Published:** 2022-09-23

**Authors:** Jie Huang

**Affiliations:** The First People’s Hospital of Linping District, Linping, China

**Keywords:** Alzheimer’s disease, positron emission tomography, neuroinflammation, mild cognitive impairment, review

## Abstract

Alzheimer’s disease (AD) is a devastating neurodegenerative disease with a concealed onset and continuous deterioration. Mild cognitive impairment (MCI) is the prodromal stage of AD. Molecule-based imaging with positron emission tomography (PET) is critical in tracking pathophysiological changes among AD and MCI patients. PET with novel targets is a promising approach for diagnostic imaging, particularly in AD patients. Our present review overviews the current status and applications of *in vivo* molecular imaging toward neuroinflammation. Although radiotracers can remarkably diagnose AD and MCI patients, a variety of limitations prevent the recommendation of a single technique. Recent studies examining neuroinflammation PET imaging suggest an alternative approach to evaluate disease progression. This review concludes that PET imaging towards neuroinflammation is considered a promising approach to deciphering the enigma of the pathophysiological process of AD and MCI.

## 1 Introduction

Alzheimer’s disease (AD) is a neurodegenerative disease with a concealed onset and continuous progress. It is a severe risk factor that is threatening the health and life of elderly individuals (1, 2). AD is known to accumulate two different insoluble protein aggregates. During the prodromal phase of AD, also called mild cognitive impairment (MCI), patients have a receding performance of cognitive domains (3). The neuropathological biomarkers for AD and MCI are β-amyloid (Aβ) plaques and intracellular tau neurofibrillary tangles (NFTs) (4, 5). Aβ cascade hypothesis is one of the dominant hypotheses of AD pathogenesis (6). It refers to the Aβ patch as the initial factor causing excessive phosphorylation of tau protein, microglial activation, neurotransmitter disorders, oxidative stress, and neuro-pathological changes (7). However, AD pathological changes may have been maintained for decades before symptoms occur. When the exact diagnosis has been made with the above approaches, patients may present with irreversible nervous system damages. Therefore, developing an early, non-invasive, and accurate diagnostic method could be a research hotspot.

The central nervous system (CNS) degeneration and disease neuropathology predate AD and MCI (8). One dominant theory indicates that the excess generation or disordered clearance of Aβ is the dominant event that initiates AD pathogenesis. However, about 30% of healthy individuals with Aβ deposition are without clinical symptoms. Nevertheless, most clinical studies revealed that high Aβ levels are associated with the severity of cognitive impairment. The hyperphosphorylated tau-based NFT pathology is positively associated with the severity of cognitive symptoms (9). However, anti-amyloid interventions have been reported to depict limited effects in clinical trials. Because of poor understanding of the pathogenesis, the clinical diagnosis of AD mainly depends on detailed medical history, imaging, and neuropsychological scale assessment. Therefore, besides the traditional biomarkers of AD pathology, more targets require further investigation in exploring AD.

Positron emission tomography (PET) is a neuroimaging approach to evaluate the molecular processes in the brain, effective and accurate for diagnostic purposes, clinical strategy planning, and assessing disease progression (10, 11). PET radioligands can bind to responding targets, including receptors, transporters, or enzymes. Furthermore, tracer binding or uptake degrees can serve as a quantitative neuropathology approach. With the advance of PET technology, targeted molecular probes toward Aβ deposition, tau protein, and neuroinflammation have been extensively developed and utilized in the clinical management of AD. PET biomarkers have also been recommended to ameliorate diagnostic accuracy for AD and MCI, including cerebral metabolism or Aβ deposition on amyloid PET (12). However, the rate of cerebral metabolism cannot explicitly elucidate potential neuropathology. Although measurement of amyloid and neuroinflammation through PET tracers provides excellent insight into the underlying process of AD and MCI, the latter has not been implemented in clinical practice. The present review describes the associated trials of PET imaging targeting neuroinflammation within AD and MCI.

## 2 AD and neuroinflammation

Currently, neuroinflammation in AD has been well illustrated (13). In the initial phase of neuroinflammation, immune cells aim to ameliorate neuronal injury. However, abnormal inflammatory responses involving prolonged microglial activation presented adverse effects and exacerbated neurodegeneration (14). According to histopathological studies, activated microglia localize to Aβ plaques and NFTs, which attached significant attention to the role of neuroinflammation in the AD process (15). Microglia are dominant immune cells in the central nervous system (CNS), essential in maintaining hemostasis and secreting inflammatory factors (16, 17). Under the hemostatic condition, microglia present M1-like status, while under pathological conditions, they convert to an M2 state (18). M1 microglia are pro-inflammatory and produce reactive oxygen species (ROS) to remove the foreign substance and trigger neuroinflammation, while M2 microglia present an anti-inflammatory function to protect the neurons. Additionally, reactive astrocyte is also a critical member of neuroinflammation, which precipitates both Aβ and tau and is closely linked to microgliosis (19, 20). Astrocytes can be classified into A1 and A2 subtypes based on their phenotype and genetic expression profiles (21). A1 astrocyte secretes and produces various inflammatory factors and neurotoxins, whereas A2 astrocyte produces neurotrophic substances (22, 23).

Neuroinflammation is considered to have an essential role in AD. It has been demonstrated that the persistent accumulation of Aβ levels in AD patients leads to activated neuroinflammation and elevated ROS, which induces cell death through apoptosis (7). Moreover, neuroinflammation initiated by infection has been revealed to exacerbate the tau pathological process in transgenic rodent AD models. Because elderly individuals are more prone to infections, the elevated Aβ burden with activated neuroinflammatory insult may accelerate the progression of neurodegeneration in patients at risk for AD (24). According to the above discussion, it is urgent to make international efforts to develop novel radiotracers for imaging neuroinflammation in AD patients with PET. Therefore, the following sections will briefly review the different strategies within advanced PET radiotracers targeting the potential molecules in the neuroinflammation process.

## 3 Application of PET imaging agents for neuroinflammation

The AD-associated neuroinflammatory biomarkers are divided into (1) enzymes or signal molecules, including 18-kDa transporters (TSPO), monoamine oxidase B (MAO-B), imidazoline binding sites I2 (I_2_BS), epoxidation enzyme, and arachidonic acid (AA); (2) G protein-coupled receptors, including purine P2X7 and P2Y receptors, and type 2 cannabinoid receptors (CB2R). Therefore, the corresponding PET tracers are developed and will be summarized in the following section.

### 3.1 TSPO PET radiotracer

It is reported that TSPO presents a variety of cellular functions, including cholesterol transport, inflammatory responses, and hormone synthesis. However, its exact role in the brain immune reaction is not fully understood. Under the hemostatic condition, the expression of TSPO maintains a low level in microglia within CNS, while after neuroinflammation, abnormal activation of microglia is associated with a high level of TSPO. Furthermore, upregulation of TSPO and activation of microglia colocalize spatially after a neurotoxic intervention was established through immunohistochemistry staining, suggesting that TSPO can detect activated microglia and can be a novel approach to measure neuroinflammation. Therefore, combining imaging agents with TSPO can serve as microglia activation and neuroinflammation biomarkers. ^11^C-PK11195 presents a high affinity to TSPO, the first classical probe used for PET neuroinflammation imaging. Accumulative evidence of PET images revealed that the region of the cingulate, temporoparietal cortex, and amygdala in patients with AD presented a high level of 11C-PK11195 absorption compared to healthy individuals. However, the low sensitivity, low bioavailability, high rate of nonspecific binding, and short half-life defects limited the application of ^11^C-PK11195 in patients with AD (25–27). Therefore, the second- and third-generation imaging agents, including the 11C-DPA713, 18F-DPA714, 18F-GE180, and 11C-ER176, exert higher affinity, and brain uptake rate can detect TSPO in neuroinflammation in low expression.

#### 3.1.1 First generation of TSPO PET radiotracers: ^11^C-PK11195

The dominant first generation of TSPO PET radiotracers was the ^11^C-PK11195, which is accompanied by either the racemic mixture or the active R-enantiomer. It has been considered the most widely used radiotracer for PET imaging in brain tissue. However, due to the intrinsic properties of the compound and complexity of carbon-11 radiolabeling within a short period of 20 min, the development of this technique for exploring neuroinflammation was impeded. Due to the poor blood–brain barrier (BBB) penetration and low brain uptake, ^11^C-PK11195 presents a poor signal-to-noise ratio. Moreover, ^11^C-PK11195 has various shortcomings, including low bioavailability and nonspecific binding, which limit its ability to determine subtle changes in TSPO expression in brain tissues (25, 27, 28). Therefore, the above difficulties challenge its application in clinical management and require further development of novel TSPO PET radiotracers.

#### 3.1.2 Second generation of TSPO PET radiotracers

A wide range of second-generation TSPO PET radiotracers have emerged, which present a higher affinity to TSPO and better characteristics. Several radiotracers have already been widely used in patients, especially in AD. According to a previous study, ^11^C-PBR28 presents a higher specific signal towards microglial activity than ^11^C-PK11195 (29). Additionally, ^11^C-DPA-713 showed better sensitivity in the healthy brain than ^11^C-PK11195 in measuring increased TSPO expression in the brain (30). Moreover, ^11^C-DPA-713 determined increased TSPO density in widespread brain regions in AD patients than ^11^C-PK11195 (31). The evaluations of TSPO radioligands, including ^11^C-DPA-713, ^18^F-DPA-714, and ^11^C-PK11195, have been compared in acute neuroinflammation rat models (32). The results indicated that ^18^F-DPA-714 had the highest ipsilateral-to-contralateral uptake ratio and had a better binding potential than ^11^C-DPA-713 and ^11^C-PK11195 (31). Moreover, the ligand ^11^C-DAA1106 has demonstrated a higher affinity than ^11^C-PK11195 to activate microglia in various neurological disorders (33, 34). The development of novel molecules with higher affinity, greater bioavailability, and the possibility of radiolabel with ^18^F facilitates the easier development of inflammation imaging. Currently, a couple of radioligands labeled with ^18^F have also been established. Among these, ^18^F-FEDAA1106 presents a higher affinity to TSPO than DAA1106. Accumulated evidence has revealed the higher brain uptake of ^18^F-FEDAA1106 than ^11^C-PK11195 or ^11^C-DAA1106 (35). Moreover, ^18^F-FEMPA could be a suitable PET radiotracer for TSPO (36). It has been described that ^18^F-FEPPA is associated with higher affinity towards TSPO, higher brain penetration, and better pharmacokinetics than the first generation of TSPO PET radiotracers (37).

#### 3.1.3 Third generation of TSPO PET radiotracers

A wide range of third-generation TSPO tracers, including ^18^F-GE-180 (R, S)-^18^F-GE-387, ^11^C-ER176, ^11^C-CB184, ^11^C-CB190, ^11^C-N′-MPB, and ^18^F-LW223, have been well established. A couple of clinical trials have compared the binding properties, brain uptake, and performance of TSPO radiotracers. The measurement of microglial activation through ^18^F-GE-180 was more sensitive than that by ^18^F-PBR06. A wide range of studies have reported on the value of ^18^F-GE-180 in assessing microglial activity in different rodent models. Furthermore, rising levels of ^18^F-GE-180 uptake indicate elevated microglial activation in patients with AD, semantic dementia, and MCI. However, another study on mouse stroke models suggested that ^11^C-DPA-713 PET presented higher accuracy and sensitivity on microglial activation measurement than ^18^F-GE-180. Moreover, a more favorable brain entrance property of ^11^C-PBR28 has also been reported compared to ^18^F-GE-180. ^11^C-ER176 has been revealed with a higher binding affinity than ^11^C-PK11195, ^11^C-PBR28, and ^11^C-DPA-71. A clinical trial of ^11^C-ER176 PET for accessing microglia activation in MCI and AD patients is still ongoing (NCT03744312).

Based on the evidence, the dominant concern for TSPO ligands is the cellular location of the signal. The different binding sites on glial and vascular TSPO have been reported for several TSPO ligands. The high-glial-TSPO-selectivity and polymorphism-sensitive ligand ^18^F-FEBMP has been developed. It presented a higher contrast to neuroinflammation than ^11^C-PK11195 in the PS19 tauopathy mouse model. Further studies assessing the binding selectivity to TSPO polymorphism among different generations of TSPO radiotracers are highly required.

### 3.2 TSPO PET imaging of neuroinflammation in AD

The following section will summarize the clinical effects secured from the clinical trials of PET imaging toward neuroinflammation among AD and MCI patients. An in-depth understanding of the underlying mechanisms of AD neuroinflammation will lead to novel therapeutic approaches to monitor disease progression using TSPO PET imaging.

#### 3.2.1 First generation of TSPO PET imaging in AD

The radiotracer has been examined in accumulative studies involving AD and MCI patients (**Table 1**). The first clinical trial focusing on ^11^C-PK11195 failed to detect TSPO binding related to microglial activation in patients with mild to moderate dementia (51). Another AD study also reported a similar result, which suggested that microglial activation presented in later stages of AD or ^11^C-PK11195 is insensitive in mild to moderate AD (40). A recent study reveals only a small cluster of significantly elevated ^11^C-PK11195 binding in occipital lobes in AD dementia patients without any difference between clinically stable prodromal AD patients and those who progressed to dementia (43). The rising ^11^C-PK11195 brain uptake among AD patients was observed in two studies: (1) higher uptake was found in the frontal and right mesotemporal regions using SPECT (52); (2) higher uptake was seen in the frontal, temporal, occipital, and striatum using PET (39). Additionally, elevated regional ^11^C-PK11195 binding is observed in the entorhinal, temporoparietal, and cingulate cortex in patients having mild and early AD. The results were consistent with another study showing increased ^11^C-PK11195 signals (38, 53)

**Table 1:** Studies examining regional brain uptake *via* first-generation TSPO tracers in AD and MCI.

Radiotracer	Included individuals	Conclusion	Year	Author
**^11^C-PK11195**	8 AD patients and 15 normal individuals	Elevated levels of radiotracer level were observed in brain areas in AD patients. Uptake in the left inferior temporal lobe differentiated AD patients with a sensitivity of 75%.	2001	Cagnin (38)
**^11^C-PK11195**	13 AD patients and 10 normal individuals	Areas in frontal temporal, parietal, and occipital association cortex showed increased radiotracer uptake in AD patients than controls.	2008	Edison (39)
**^11^C-PK11195**	6 AD patients, 6 MCI patients, and 5 normal individuals	No statistic difference in TSPO binding was observed when comparing the AD with controls in any brain region.	2009	Wileey (40)
**^11^C-PK11195**	14 MCI patients and 10 normal individuals	Frontal cortical regions presented higher TSPO binding in MCI patients compared to controls.	2009	Okello (41)
**^11^C-PK11195**	11 AD patients and 10 normal individuals	Higher ^11^C-PK11195 retention was observed in medial frontal, parietal, and left temporal cortical areas in AD patients compared to controls. Additionally, uptake in the left anterior cingulate, left precuneus, left hippocampus, and left medial frontal cortex presented negative relationship with cognitive performance.	2011	Yokokura (42)
**^11^C-PK11195**	19 AD patients, 10 MCI patients, and 21 normal individuals	The bilateral occipital cortex is the only brain region assessed with a statistical difference between AD patients and controls, while no such differences were found when comparing MCI patients to controls.	2013	Schuitmaker (43)
**^11^C-PK11195**	8 AD patients and 14 normal individuals	Increased microglial tracer uptake in frontal, parietal, occipital, temporal cortical areas, and striatum and hippocampus was observed in AD patients compared to controls.	2015	Fan (44)
**^11^C-PK11195**	10 AD patients, 10 MCI patients, and 16 normal individuals	Cortical retention of ^11^C-PK11195 in the occipital lobe, temporal lobe, hippocampus, parahippocampus, temporal, and precentral and postcentral gyrus was higher in AD patients compared to controls. Additionally, temporal, frontal, orbital, straight, parietal gyrus, insula, putamen, and occipital lobe presented higher ^11^C-PK11195 retention in MCI compared to controls.	2015	Fan (45)
**^11^C-PK11195**	8 AD patients and 8 normal individuals	^11^C-PK11195 uptake in the areas of medial temporal regions and the hippocampus in AD was negatively related to hippocampal volume.	2016	Femminella (46)
**^11^C-PK11195**	8 AD patients, 8 MCI patients, and 14 normal individuals	MCI patients showed reductions in ^11^C-PK11195 uptake in the region of temporal, occipital, parietal, cingulate cortex, and the hippocampus after 14 months, while AD patients showed an increase in microglial activation than controls.	2017	Fan (47)
**^11^C-PK11195**	42 MCI patients and 10 normal individuals	In amyloid positive MCI subjects, TSPO binding was elevated in frontal, parietal, and lateral temporal regions compared to controls. Moreover, positive correlation was observed between the results of ^11^C-PK11195 and ^11^C-PiB in frontal, temporal, and parietal brain areas.	2017	Parbo (48)
**^11^C-PK11195**	16 AD and MCI patients, and 13 normal individuals	Areas within the occipital, parietal, temporal cortex, and medial temporal regions showed increased radiotracer uptake in the AD and MCI combined group compared to controls.	2018	Passamonti (49)
**^11^C-PK11195**	6 AD patients, 20 MCI patients, and 20 normal individuals	In the areas of frontal, posterior cingulate, parahippocampal, lateral and posterior temporal cortex, precuneus, and hippocampus, increased TSPO binding was observed in MCI patients compared to controls.	2018	Parbo (50)

Moreover, two other longitudinal studies provided the course of neuroinflammation in AD *via* ^11^C-PK11195. The first study revealed an increase in radiotracer signals in AD patients (44). On the contrary, another longitudinal study illustrated the evolution of ^11^C-PK11195 in eight MCI patients, four of whom revealed negative amyloid imaging. In contrast, a longitudinal reduction of microglial activation was observed in this population (47). The small sample sizes, different methods, and first-generation TSPO radiotracer limits could elaborate on these contradictory results.

#### 3.2.2 Second generation of TSPO PET imaging in AD

The shortcomings of the second generation of TSPO radiotracers were improved with the development of advanced-generation TSPO ligands with an enhanced signal-to-noise ratio and higher binding affinity compared to ^11^C-PK11195 (54). Several studies assessed neuroinflammation with the measurement of radiotracer retention in AD patients through these advanced ligands (**Table 2**). This section will summarize the second-generation TSPO radiotracers widely used in AD or MCI. Elevated region-specific TSPO binding signals in a wide range of cortical areas are more illustrated in patients with AD than in normal individuals. The temporal pattern of neuroinflammation over the course of AD has also been well-characterized through longitudinal investigations (55, 60, 62). The role of neuroinflammation is revealed by a large longitudinal study with the administration of ^18^F-DPA-714 (60, 61). Participants with MCI and higher initial TSPO binding indicate a slower rate of decline due to dementia than those with lower initial TSPO binding. Combined with the other ^11^C-PK11195 studies, the proposal of a dual peak hypothesis of neuroinflammation in AD has been well-established (47). The hypothesis illustrated that neuroinflammation in the early phase of MCI patients is protective and beneficial to Aβ removal, while activated neuroinflammation in the later phase is detrimental. Notably, the results from TSPO imaging studies could reflect different PET signals to explore the association between TSPO expression and clinical outcomes. Similar to ^11^C-PK11195, enhanced TSPO signals are related to impairments in cognition and memory, visuospatial and language ability, executive functioning, dementia severity, and brain atrophy. Further research and clinical trials are urgently required when illustrating regional uptake patterns of TSPO ligands in MCI. Some studies indicated that striking patterns of high cortical tracer retention, especially in the temporal lobe, have been observed compared to healthy controls (47, 63, 65).

**Table 2:** Studies examining regional brain uptake *via* advanced TSPO tracers in AD and MCI.

Radiotracer	Included individuals	Conclusion	Year	Author
**^11^C-PBR28**	19 AD patients, 10 MCI patients, and 13 normal individuals	Areas of prefrontal, inferior parietal, temporal, precuneus, posterior cingulate, occipital, hippocampus, and entorhinal cortex presented higher ^11^C-PBR28 binding in AD patients compared to controls, while no such difference was observed in MCI patients.	2013	Kreisl (55)
**^11^C-PBR28**	25 AD patients, 11 MCI patients, and 21 normal individuals	Areas of temporal and parietal brain presented higher ^11^C-PBR28 uptake in AD patients compared to controls, while no such difference was observed in MCI patients.	2015	Lyoo (56)
**^11^C-PBR28**	14 AD patients and 8 normal individuals	Areas of inferior parietal lobule, occipital cortex, precuneus, entorhinal cortex, hippocampus, inferior, and middle temporal cortex presented higher ^11^C-PBR28 binding in AD patients compared to controls. Annual increase in radiotracer binding was also observed in AD patients.	2016	Kreisl (57)
**^11^C-PBR28**	13 MCI patients and 9 normal individuals	Higher radiotracer uptake in the temporal lobe, post-cingulate cortex, thalamus, medial temporal lobe, hippocampus, amygdala, and cerebellum can be observed in MCI patients than controls.	2018	Fan (58)
**^11^C-PBR28**	16 AD patients, 16 MCI patients, and 19 normal individuals	Positive correlations were found between ^1^C-PBR28 and amyloid retention on ^18^F-flutemetamol and tau aggregation measured by^18^F-AV-1451.	2018	Dani (59)
**^11^C-DPA-713; ^11^C-PK11195**	17 AD patients and 22 normal individuals	^11^C-DPA-713 presented higher accuracy in TSPO binding than ^11^C-PK11195 in AD patients, and demonstrated an inverse relationship with cognition.	2017	Yokoura (31)
**^18^F-DPA-714**	64 AD patients and 32 normal individuals	Areas of precuneus, parietal, temporal cortex, and medium and posterior cingulate presented higher ^18^F-DPA-714 uptake in AD patients compared to controls.	2016	Hamelin (60)
**^18^F-DPA-714**	52 AD patients and 17 normal individuals	Areas of temporal and parietal brain presented higher tracer retention in AD patients relative to controls. Annual increases of 13.2% were observed for AD patients.	2018	Hamelin (61)
**^11^C-DAA1106**	10 AD patients and 10 normal individuals	Areas of cerebellum, prefrontal cortex, parietal cortex, temporal cortex, occipital cortex, anterior cingulate cortex, and striatum presented higher ^11^C-DAA1106 binding in AD patients compared to controls.	2008	Yasuno (62)
**^11^C-DAA1106**	10 AD patients, 7 MCI patients, and 10 normal individuals	Areas of striatum, lateral temporal, parietal, and anterior cingulate cortex presented increased ^11^C-DAA1106 binding in AD patients compared to controls.	2012	Yasuno (63)
**^18^F-FEPPA**	21 AD patients and 21 normal individuals	Areas of temporal, frontal, parietal, and occipital cortical regions, and the hippocampus presented increased ^18^F-FEPPA retention in AD patients compared to controls.	2015	Suridjan (64)
**^18^F-FEPPA**	11 MCI patients and 14 normal individuals	A positive correlation between ^18^F-FEPPA binding and ^11^C-PiB can be observed in aMCI in the hippocampus.	2017	Knezevic (65)
**^18^F-FEMPA**	10 AD patients and 7 normal individuals	Areas of medial temporal, lateral temporal, and posterior cingulate cortex, putamen, caudate, thalamus, and cerebellums presented increased ^18^F-FEMPA uptake in AD patients compared to controls.	2015	Varrone (36)
**^18^F-GE-180**	6 AD patients and 7 normal individuals	Cerebellum is a suitable pseudo-reference region for PET imaging of AD by ^18^F-GE-180. No significant increases in ^18^F-GE-180 binding in the frontoparietal VOIs of patients with AD when compared to the healthy controls.	2021	Vettermann (66)

Nevertheless, it is necessary to understand the limitation of second-generation TSPO radiotracers, particularly sensitivity to the single-nucleotide polymorphism (SNP) of the TSPO gene. Genetic variation in the SNP rs6917 leads to different binding patterns in TSPO. Therefore, it is critical to evaluate these genetic polymorphisms when examining data from these radiotracers and exclude low-affinity binders (54, 67). Consequently, the advantage of ^11^C-PK11195 can be emphasized without any influence of TSPO polymorphism.

### 3.3 Other radiotracers targeting neuroinflammation

It is imperative to develop other novel and effective imaging radiotracers to measure neuroinflammation with higher specificity and affinity since TSPO is not specifically located in microglia. Therefore, promising targets should present precise localization in neuroinflammation and particular ligands to enable imaging measurement (68–71). Further work is required to explore novel targets for the activated microglia and their ability to phagocytose Aβ. Transcriptional profiling of human microglia in plaque-associated and parenchymal tissues could decipher changes in whole tissue RNA, leading to novel targets identified and prioritized for further investigation (72). In the following section, we summarized the other neuroinflammation-targeted ligands for imaging neuroinflammation.

#### 3.3.1 CSF1R PET imaging

Colony-stimulating factor 1 receptor (CSF1R) is dominantly expressed in the microglia, macrophages/monocytes, and dendritic cells in the brain parenchyma. CSF1R presents an essential role in microglia growth, proliferation, and survival. Previous studies have determined that the growth factors of colony stimulating factor-1 (GCSF1) and interleukin-34 (IL-34) are endogenous ligands toward CSF1R (73). It has also been demonstrated that the upregulation of CSF1R is responsible for injury and AD-related neuropathology (74, 75). Due to the role of CSF1R, a novel CSF1R-targeting radiotracer ^11^C-CPPC was developed. In animal acute inflammation models, the encephalomyelitis model of multiple sclerosis, and APPsi with cerebral Ab pathology, increased microglial levels of CSF1R have been captured through this radiotracer (76). Moreover, a recent study compared novel CSF1R tracers ^11^C-GW2580 with ^11^C-CPPC in detecting both acute inflammation induced by LPS injection and chronic inflammation in APP knockin mice. The results revealed that ^11^C-GW2580 signal changes in CSF1R showed higher sensitivity than ^11^C-CPPC, associated with increased TSPO pattern in the brain (77).

#### 3.3.2 COX1 and COX2 PET imaging

Cyclooxygenase (COX) plays an essential role in the generation of prostaglandin H2, the substrate for prostaglandins and thromboxanes. There are two isoforms of COX, COX-1 and COX-2, determined to present a critical role in neuroinflammation and links to various neurodegenerative diseases, especially AD. According to the results of immunochemical evidence, COX-1 and COX-2 are located in both microglia and neuron in the CNS (78). Various radiotracers for COX-1 and COX-2 have been well established, such as ^18^F-TMI, ^18^F-triacoxib, ^11^C-rofecoxib, ^11^C-KTP-Me, ^11^C-PS13, and ^11^C-MC1 (79–86). A few studies demonstrated that ^11^C-KTP-Me harbors a greater BBB entrance and selective sensitivity towards COX-1 (80, 86). Moreover, clinical trials with ^11^C-KTP-Me revealed an elevated brain signal in AD patients compared to normal individuals. ^11^C-KTP-Me accumulation can be seen in activated microglia surrounding Aβ plaques within the frontal cortex and hippocampus. Similar *in vivo* studies can also be observed in APPswe (Tg2576) mice compared to wild-type mice (80, 81). Additionally, previous studies illustrated that both ^11^C-PS1 (COX-1 PET imaging) and ^11^C-MC1 (COX-2 PET imaging) radiotracers showed specific detection patterns after LPS-induced neuroinflammation in monkey brain and human inflammatory tissues (82, 83).

#### 3.3.3 P2X7R and P2Y12R PET imaging

The upregulated levels of the purinergic P2X7 receptor (P2X7R) can activate neuroinflammation, especially in M1 microglia. P2X7R had various biological functions, including inflammasome activation, cytokine secretion, T lymphocyte differentiation, and cell death (87). Microglia monitors through P2Y12R-dependent junctions are associated with mitochondrial activity in neurons (88). Brain injuries altered somatic junctions that induced P2Y12R-dependent neuroprotective effects through calcium load and functional connectivity in neurons (89, 90). Based on the immunohistochemical staining results, levels of P2Y12R were decreased in the brains of AD patients (91). Accumulative evidence has revealed that various P2X7R-targeting radiotracers have been developed currently, such as ^11^C-GSK1482160, ^11^C-JNJ-47965567, ^18^F-JNJ-64413739, ^11^C-JNJ-54173717, ^11^C-SMW139, and ^18^F-PTTP (92–98). A previous study overexpressed human P2X7R in a rat model by rAAV3flag-hP2X7R and demonstrated that ^11^C-SMW139 had higher affinity and specificity to the P2X7R (99). Moreover, brains of AD patients revealed higher ^11^C-SMW139 binding compared to healthy individuals through autoradiography, consistent with histological staining results (99). An ongoing clinical trial applied ^11^C-SMW139 as a PET imaging radiotracer toward neuroinflammation in Parkinson’s disease (PRI-PD: 2018-000405-23). The other probes are P2Y12R-based radiotracers, including ^11^C-AZD1283, ^11^C-P2Y12R-ant, and ^11^C-5, assessed among animal models (94, 100, 101). A previous study has demonstrated that the P2Y12R radiotracer, ^11^C-AZD1283, distinctly responds to tau and amyloid deposits. The levels of P2Y12R binding increase in APP23 and APP^NL-F/NL-F^ mice (101). However, ^11^C-AZD1283 PET imaging showed no signal in the wild-type mouse brain.

Additionally, two other radiotracers, ^11^C-P2Y12R and ^11^C-5, exerted sufficient brain uptake, high affinity, and promising results in experimental autoimmune encephalomyelitis and stroke models, detecting anti-inflammatory microglia (48) (94, 100).

#### 3.3.4 MAO-B PET imaging

In several clinical trials, ^11^C-deuterium-L-deprenyl (DED) MAO-B inhibitors have also been applied in PET imaging toward neuroinflammation. It has been proved that early astrocytosis can be measured *via* ^11^C-DED in sporadic and autosomal dominant AD patients and amyloidosis mouse models (102–110). In addition, ^18^F-fluorodeprenyl-D2 showed favorable kinetic properties and ameliorated affinity in MAO-B imaging (111). However, the technical problems of irreversible inhibitors impede the accuracy of imaging. Therefore, several reversible-binding inhibitors have been developed and validated, including ^11^C-Cou, ^11^C-SL25.1188, and ^11^C-SMBT-1 (69, 112, 113). Among the advanced PET imaging based on reversible-binding inhibitors, a specific elevated regional retention of ^11^C-SMBT-1 within the cortical and hippocampal regions can be seen in patients with AD compared to healthy individuals (114).

#### 3.3.5 I_2_BS PET imaging

I_2_BS is located on both monoamine oxidases A (MAO-A) and B (MAO-B), which is another novel target for PET imaging toward neuroinflammation (115–118). ^11^C-FTIMD presented the specific binging to I₂BS in the monkey brain (119). A previous study has revealed that the activated astroglia determined using ^11^C-BU99008 PET in the early period of Parkinson’s disease is responsible for α-synuclein accumulation (116). A previous *in vitro* study demonstrated that ^3^H-BU99008 revealed high specificity in brain tissues from AD patients and colocalized with glial fibrillary acidic protein staining of astrocytes (120). Moreover, a clinical study reported that an increasing ^3^H-BU99008 binding signal could be detected in the brain of patients with AD compared to healthy individuals. Similar results were reported in the cortical region *via* 11C-BU99008, consistent with the high cerebral Aβ load evaluated by ^18^F-florbetaben in MCI and AD patients (121). A previous study demonstrated that increased ^11^C-BU99008 signaling could be detected in earlier stages with low Aβ loads. At the same time, reduced astrocytosis can be observed in the advanced stages with a more significant Aβ load and atrophy (122).

#### 3.3.6 CB2R PET imaging

Cannabinoid type 2 receptor (CB2R) is a member of the endogenous cannabinoid system. CB2R has a low concentration in the brain during healthy conditions. However, Aβ deposition activates microglia leading to the high expression of CB2R, another widely considered dominant marker of AD neuroinflammation. Various PET radiotracers with higher affinity towards CB2R have been well established. Among the developed CB2R imaging agents, ^11^C-NE40 can specifically and reversibly bind to CB2R. Previous studies have depicted that the uptake of ^11^C-NE40 was reduced in the brain of AD patients due to loss of neuron-based CB2R expression, contrary to the expectations from preclinical studies. This inconsistency may be due to the low expression of CB2R and insufficient selectivity for CB2R and CB1R (123). The other CB2R agonists with high affinity are under exploration, including ^11^C-MA2, ^18^F-MA3, or ^18^F-RS126 (124).

#### 3.3.7 ^11^C-AA PET imaging

AA, a kind of n-6 polyunsaturated fatty acid and an essential component of the metabolic network of inflammation, is abundant in the brain parenchyma and participates in cell signal transduction. Microglia in the CNS release the inflammatory cytokines and bind to receptors on the surface of astrocytes, leading to the secretion of phospholipase and cytoplasmic phosphatase and mediated AA release. Therefore, AA detection can serve as an indirect marker of microglial activation (125). Based on the above results, 11C-AA and PET imaging can determine brain phospholipase activity. By injecting ^11^C-AA, the regional brain incorporation coefficients and metabolic loss of AA in the brain can be determined (125). Therefore, elevated signals of ^11^C-AA could evaluate the disordered metabolism of AA due to neuroinflammation.

#### 3.3.8 Nicotinic acetylcholine receptors

It has been demonstrated that nicotinic acetylcholine receptors (nAChRs) were closely related to neuroinflammation. Moreover, the ligand 2-18F-A85380 (2-FA) towards nAChR has also presented similar patterns of uptake with 11C-PK11195 in activated microglia and astrocytes (126). Additionally, 18F-flubatine has been established with more favorable kinetic profile, which leads to a better understanding of nAChRs in neuroinflammation (126, 127). The homomeric nAChRs are colocalized in neuritic plaques in patients’ brain with AD, and Aβ1–42 has been reported to bind to the α7 nAChRs with high affinity (128). α7 nAChRs are also strongly expressed on astrocytes and microglia, the activation of which has been shown to suppress inflammatory processes (128). Recently, several new compound-based bio-tracers, such as 18F-ASEM and 18F-DBT-10, presented more promising results (129).

## 4 Methodological issues in radiotracers quantification by PET

A variety of quantification methods have been established due to the tracers. It appears that it is a great challenge for quantification by PET and that various factors should be taken into account. (1) Genetic polymorphism and affinity: The main limitation of the second-generation TSPO radiotracers is their sensitivity to a polymorphism of the TSPO gene. This polymorphism leads to differential affinity of these ligands to TSPO (67). (2) Arterial plasma input function: The kinetics model analyses are based on the input function, which requires the relative traumatic placement of an arterial catheter and the development of radioanalytical methods to accurately identify the plasma metabolite fractions (56). (3) Reference region definition: The reference region should be devoid of specific ligand binding to the target and only share the same free and non-specific binding with the region expressing the target and remain unaffected by the disease.

## 5 Conclusion

The interplay between amyloid, tau, and neuroinflammation is a brand new area of investigation that has only recently become possible through the development of an expanded repertoire of PET tracers. Therefore, we reviewed various strategies for PET imaging for neuroinflammation and summarized the dominant results from previous clinical trials on AD patients. There are a variety of approaches explaining the exploration of neuroinflammation. Although determining neuroinflammation by PET imaging in AD is widely used, there is a conflict on whether neuroinflammation is beneficial or detrimental to the evolution of the symptoms, neuronal injury, and cognitive deficits. This review also highlighted are a number of areas of uncertainty and various radiotracers’ limitations. The challenges of radiotracer establishment and accurate binding quantification, the complicated neuroinflammation in the brain tissue of AD patients, and its extensive roles in different stages have also been emphasized. PET imaging towards neuroinflammation is a potential approach to deciphering the pathophysiological process of AD patients. It clarifies the links between amyloid/tau pathologies and neuroinflammation, influencing different clinical symptoms and pathophysiological progression. More efforts are also required to improve the approaches to determine the binding signal from the PET imaging based on the current radiotracers and develop novel radiotracers. Moreover, deciding on a consensus to standardize the PET data analysis is critical. Given the proposal that the role of neuroinflammation in AD pathogenesis changes over the disease course, this is a prerequisite to conducting multi-tracer longitudinal studies based on multiple centers and elucidating the multifaced role of neuroinflammation in AD and MCI patients.

## Author contributions

The author confirms being the sole contributor of this work and has approved it for publication.

## Conflict of interest

The author declares that the research was conducted in the absence of any commercial or financial relationships that could be construed as a potential conflict of interest.

## Publisher’s note

All claims expressed in this article are solely those of the authors and do not necessarily represent those of their affiliated organizations, or those of the publisher, the editors and the reviewers. Any product that may be evaluated in this article, or claim that may be made by its manufacturer, is not guaranteed or endorsed by the publisher.
